# Mating structure of the blue and red shrimp, *Aristeus antennatus* (Risso, 1816) characterized by relatedness analysis

**DOI:** 10.1038/s41598-019-43523-w

**Published:** 2019-05-10

**Authors:** Laia Planella, Manuel Vera, Jose-Luis García-Marín, Sandra Heras, María Inés Roldán

**Affiliations:** 1 Laboratori d’Ictiologia Genètica, Universitat de Girona, Girona, Spain; 2 Present Address: Departamento de Zooloxía, Xenética e Antropoloxía Física, Campus Lugo, Universidade de Santiago de Compostela, Lugo, Spain

**Keywords:** Ecological genetics, Animal behaviour

## Abstract

Understanding life history variation and strategies is crucial for stock assessment and fisheries management due to the direct effects on population dynamics, effective population size, sex-ratios, levels of inbreeding, and relatedness among individuals. *Aristeus antennatus* (En ─ Blue and red shrimp; Fr ─ Crevette rouge; Sp ─ Gamba rosada) is one of the most exploited demersal resources in the Western Mediterranean Sea. However, information regarding the mating system and mate choice preferences remains largely unknown. Advances in molecular genetic markers and methods of inferring biological relationships among individuals have facilitated new insights into the reproductive dynamics of the species in the wild. Here, we used microsatellite markers to examine the *A*. *antennatus* mating system and putative mate choice preferences. Our results provided clear evidence of polyandry and polygyny. Relatedness analyses, together with *F*_ST_ and DAPC values showed females exhibited a mating bias towards unrelated males. Mating males were inferred from spermatophores and suggested males were sympatric with females and were also from other spawning grounds. Our findings provided the first description of the reproductive behavior of blue and red shrimp.

## Introduction

Mating systems have numerous implications for the ecology and evolution of species due to the effects on population dynamics, effective population size (*Ne*), levels of inbreeding, and relatedness among individuals^1,2^. It is crucial to elucidate life history variation and reproductive strategies in exploited marine species for accurate stock assessment and sustainable fisheries management^3^ and references therein. A variety of basic mating patterns are described for crustaceans updated in^4^, including exploited marine species. These studies have often focused on reproductive morphological traits, size and age at first maturity, and fecundity or spawning frequency^3,5^. Less attention has been devoted to social interactions, mating systems, mate choice, or sex-biased dispersal^3,5^. In addition, the reproductive biology of deepest sea taxa is poorly elucidated because the nature of the habitat imposes technical difficulties in observing mating behaviour^6^. Moreover, studies under captivity conditions might alter mating behaviors or kinship structure^7^.

The mating system might be simply described as the number of mates that individuals have during the breeding season^8^. Besides this basic definition, Asakura^8^ described mating systems in terms of the genetic relationships between mating individuals. Barbosa *et al*.^9^ reported that the reproductive strategy was toward improving offspring production and viability. Research has long demonstrated inbreeding caused by the mating of related individuals results in lower offspring survival and an overall population decline updated in^10^. Dispersal and mate choice reduce mating between closely related individuals^11^, but mate choice based on kin recognition is the typical pattern in invertebrate species. For example, females of the German cockroach (*Blattella germanica* Linnaeus, 1767) mate preferentially with distantly related males to increase their reproductive success^12^. However, Loyau *et al*.^13^ reported inconsistent results in *Drosophila melanogaster* (Meigen, 1830), where a reduction of offspring viability was observed when mating occurred between unrelated individuals. Such outbreeding depression resulted from breaking apart co-adapted beneficial gene complexes or local genetic adaptations^13^.

Weir *et al*. reviewed in^14^ defined relatedness between individuals as the probability of a set of genes shared between individuals are identical-by-descent (IBD). Measuring relatedness requires known pedigrees, but often pedigree information is not available in wild populations^14^. Recent advances in molecular markers facilitate probability estimates of shared ancestry from observed genotypes of a set of loci, and several Maximum Likelihood (MLE) and Method of Moment (MoM) estimators have been developed^15^. The ideal situation is when allele frequencies are known from the ancestral population^14^. Nevertheless, this is again an unrealistic situation for most wild populations and biased estimates are therefore obtained^14,16^. In any case, methods inferring relatedness between individuals within and among populations have thoroughly facilitated our understanding of wild populations^16^. Relatedness estimates serve to address several important conservation questions, e.g. mating behaviors, genetic structure, or dispersal dynamics^14^. For example, Stow *et al*.^17^ demonstrated relatedness due to isolation-by-distance, high female philopatry to their natal nest, and polygynandrous mating in the Australian allodapine bee (*Exoneura nigrescens* Friese, 1899). Kümmerlu and Keller^18^ conducted relatedness analyses and showed limited queen and free adult worker dispersal in a population of the ant *Formica exsecta* (Nylander, 1846)^18^. In marine crustacean species, few studies of mating preferences related to kinship have contributed to our understanding of population dynamics^19^, but studies in open thelycum (absence of seminal receptacles) shrimp species not suitable to laboratory conditions have not been published.

*Aristeus antennatus* (Risso, 1816) (En ─ Blue and red shrimp; Fr ─ Crevette rouge; Sp ─ Gamba rosada) is one of the most exploited demersal fisheries resources in the Western Mediterranean Sea^20^. However, information regarding the mating system and mate choice preferences remain largely unknown. The species inhabits the Mediterranean Sea at depths ranging from 80 to 2800 m^21^, with sex and size spatial and temporal segregation^22^. Adult females are primarily distributed at shallower depths between 400 and 1000 m throughout the year, while adult males and juveniles inhabit deeper zones below 1000 m^23^. In the Western Mediterranean Sea, reproduction occurs from early spring to summer, when mature males and females form spawning aggregations at depths between 600–800 m^23^ and references therein. The female-biased sex ratio in these aggregations (females may reach up 80% of catches)^23^, suggests some degree of polygyny in *A*. *antennatus*. During mating, males place spermatophores in the females’ external genitalia (thelycum) with specialized enlarged coupled and folded endopods of the first pair of pleopods, which constitute the copulatory organ called the petasma^24^. Females possess no seminal receptacles; hence, spermatophores attached in the thelycum are the only way to store sperm^24^. In other decapod species, spermatophores are lost at each molt^25^, so the high molt activity observed in *A*. *antennatus* females from May to September suggests mating might occur several times each reproductive season^26^. After mating, the shrimp shoal breaks up and males return to submarine canyons, while females remain in shallower waters carrying one or more spermatophores in the thelycum^27^. More details on how fertilization occurs remain unknown, but undoubtedly it happens in open waters and parental care is absent^27^, which complicates mating system determination.

In this study, we examine the mating system dynamics and genetic diversity of *A*. *antennatus* using microsatellite loci. In particular, we assessed multiple mating rates, male origin of multiple spermatophores attached to female thelycum, and putative mate choice preferences. These data will provide the first insights into mating systems and reproductive behaviors of blue and red shrimp, which is crucial to understand how exploitation influences the resistance of this species to catastrophic decline and subsequent resilience.

## Materials and Methods

### Sampling and DNA extraction

In August 2015, a total of 111 samples of mature *A*. *antennatus* adults (59♂: 52♀) were collected while on board the *Nova Gasela* trawling vessel at the spawning ground of Palamós (600 m depth), Spain (41°53′040″N and 3°23′777″E). Mature males were identified based on rostrum shorter than 12 mm in length^28^ and the presence of petasma fusion^24^. Similarly, we followed Sardà and Demestre^29^ to identify mature females: the cephalothorax was larger than 33 mm and one or more spermatophores were located in the thelycum. Individuals were quickly transported to the laboratory on ice to remove all the spermatophores from the females (61 spermatophores). Each spermatophore was stored separately in a 1.5 mL Eppendorf at −20 °C to extract DNA using the differential lysis protocol by Planella *et al*.^30^ to attempt to genotype males successfully mating with females. In addition, a piece of muscle tissue from each adult individual was preserved in 95% ethanol until DNA extraction was performed using the genomic DNA extraction protocol outlined in Fernández *et al*.^31^.

### Microsatellite loci

Genetic diversity at thirteen polymorphic microsatellite loci developed for *A*. *antennatus*^32^ were analyzed using one singleplex and three multiplex PCR panels delineated to avoid primer-dimer and hairpin formation (assessed *in silico* with AUTODIMER v 1.0 program^33^). For each locus, the forward primer was labeled with one fluorescent dye (PET, 6 - FAM, NED or VIC) and loci with overlapping allelic ranges were tagged with different dyes. Multiplex PCRs were conducted in a 10 µL final volume containing 1X GoTaq® G2 Hot Start Colorless Master Mix (Promega Corporation), primer multiplex mix at primer-specific concentrations (Table 1), 0 or 1.5 mM MgCl_2_ solution (adults and spermatophores, respectively), and ~40 ng of template DNA. The Aa1255 locus was analyzed in a singleplex panel performed in a 15 µL total reaction mixture containing 1X NH_4_ Reaction Buffer, 1.5 or 4.6 mM MgCl_2_ solution (adults and spermatophores, respectively), 1 mM of dNTP, 0.2 mM of each primer (forward and reverse), 0.375 units BIOTAQ^TM^ DNA Polymerase (Bioline), and ~40 ng of template DNA. All PCR reactions proceeded as follows: initial denaturation step of 2 min at 94 °C, followed by 30 (multiplex) or 35 (singleplex) cycles of 30 s at 94 °C; annealing for 1 min 30 s at 50 °C or 60 °C; and elongation for 1 min 30 s at 72 °C; with a final extension of 30 min at 60 °C (Table 1). One µL of PCR product was mixed with 0.15 µL of GeneScan^TM^ 500LIZ Size Standard (Applied Biosystems) and 10 µL of highly deionized (Hi-Di) formamide (Applied Biosystems) and denatured for 3 min at 95 °C. Resulting amplicons were run on an ABI PRISM 3130 Genetic Analyzer (Applied Biosystems) and genotypes scored using GENEIOUS R7.1^34^.

**Table 1:** Characteristics of the singleplex and three multiplex PCR reactions used to amplify 13 microsatellite loci in *A*. *antennatus*.

	Locus	Annealing temperature (°C)	Fluorescent dye 5′- forward	Allele size range	Primer concentration (µM)ª	GenBank accession number
SP	Aa1255	50	VIC	115–163	0.2	KU195295
MP 1	Aa138	50	6-FAM	187–249	0.1	KU195272
	Aa496b	50	PET	412–418	0.05	KU195279
	Aa956	50	VIC	193–217	0.1	KU195289
MP 2	Aa123	60	6-FAM	425–435	0.1	KU195269
	Aa667	60	NED	241–265	0.1	KU195282
	Aa681	60	VIC	224–322	0.1	KU195283
	Aa751	60	PET	225–235	0.1	KU195285
	Aa1444	60	PET	180–202	0.4	KU195297
MP 3	Aa421	60	PET	169–241	0.1	KU195278
	Aa818	60	6-FAM	160–210	0.2	KU195287
	Aa1061	60	VIC	143–211	0.2	KU195290
	Aa1195	60	NED	189–204	0.2	KU195293

SP, singleplex; MP1, multiplex 1; MP2, multiplex 2; MP3, multiplex 3; ªPCR final volume primer concentration.

### Diversity within and between sexes

Genetic diversity levels within sexes (females and males) were estimated as the number of alleles per locus (*N*_A_) and the observed (*H*_O_) and expected (*H*_E_) heterozygosity. In addition, at each sex and locus, the conformance of genotypic proportions to Hardy-Weinberg expectations (HW) was calculated using the program FSTAT v 2.9.3.2^35^ and summarized using Wright’s fixation index (*F*_IS_). Loci exhibiting significant positive *F*_IS_ values were evaluated for potential genotyping errors due to stuttering, allele dropout, or null alleles using MICRO-CHECKER v 2.2.3 software^36^. The Dempster *et al*.^37^ algorithm was employed to estimate the frequency of null alleles; however modifications were implemented in the FREENA software^38^. Genetic diversity differences (*N*_A_, *H*_O_, *H*_E_, *F*_IS_) between sexes were statistically tested in SPSS v 23^39^ using the Wilcoxon signed rank test. Genetic differentiation between males and females was examined by *F*_ST_ values using the program FSTAT v 2.9.3.2^35^.

Spermatophores indicated mature males effectively mating with females; therefore, we also examined these individuals for deviations from HW expectations and diversity levels, as detailed above for males and females. Diversity levels for the spermatophore group were compared with males and females and genetic differentiation from these two groups was estimated as *F*_ST_ values. Furthermore, discriminant analysis of principal components (DAPC) using the package ADEGENET v 1.4–2^40^ was performed in R v. 3.3.2^41^ to establish an additional view of differentiation among the three groups (males, females and spermatophores). DAPC does not require any specific population structure model and it is free of assumptions regarding Hardy-Weinberg or gametic equilibrium^42^. Alleles are considered original variables with the largest between-group and smallest within-group variance.

### Paternity and relatedness in the spawning ground

The exclusion probability of identity (*PI*) (probability that two randomly selected individuals matched their genotypes by chance) was applied to test the potential that the set of microsatellite loci was robust for individual identification using the software GIMLET v 1.3.3^43^. The regrouping genotype option was chosen to detect multiple paternity by comparing all spermatophore genotypes. This analysis was conducted to detect males mating multiple times with either the same female or different females. Similar comparisons were used to ascertain if some spermatophores originated from sampled males.

Relatedness analyses were used to determine if all specimens (females, males, and mating males inferred from spermatophores) in the spawning ground were derived from the same population. We first confirmed the efficiency of seven relatedness estimators available in COANCESTRY v 1.0.1.7 software^44^. We subsequently applied the related estimator to distinguish among low relatedness groups, which might be inherent in this species^45^; 1,000 dyads were simulated for each of the following relationships: first cousins (expected relatedness: *r*_xy_ = 0.125), second cousins (*r*_xy_ = 0.031), and unrelated (*r*_xy_ = 0). The best estimator was chosen for additional relatedness comparisons. Consequently, the distribution of observed pairwise relationship coefficients within each reproductive group (male, female, and spermatophores) was compared using COANCESTRY v 1.0.1.7^44^ with the relationship coefficients expected for a sample of 1,000 unrelated simulated offspring generated by mating 1,000 simulated males and 1,000 simulated females from our male and female genotypes. All simulated individuals were obtained using HYBRIDLAB v 1.0 software^46^. In addition, comparisons of relatedness between all male, female, and spermatophore pairs were used to determine if all sampled individuals derived from the same population. Finally, average relatedness in observed female-spermatophore pairs was compared to all other potential female-spermatophore pairs (excluding observed female–spermatophore pairs) and female-male pairs to determine if mate choice was associated with kinship. For each of the above comparisons, 1,000,000 replicate iterations were computed in COANCESTRY v 1.0.1.7^44^ and a genotyping error rate of 2% was allowed.

## Results

### Diversity analysis

All loci were polymorphic for each studied reproductive group (females, males, and spermatophores), with the number of alleles ranging from 2 to 19 (Table 2). Average observed heterozygosity (*H*_O_) ranged from 0.3939 in adult females to 0.4668 in mating males, genotyped from the 61 spermatophores. In the three groups, larger average *H*_E_ compared with *H*_O_ was detected, ranging from 0.6116 in females, 0.6166 in males, and 0.6190 in spermatophores. Significant differences were not detected for these three diversity measures between males and females, but the Wilcoxon signed rank test indicated the average *N*_A_ (*Z* = 2.072; *P* = 0.038) and *H*_O_ (*Z* = 2.132; *P* = 0.033) were larger in spermatophores than in females. Several loci for each reproductive group showed significant positive *F*_IS_ values and *H*_O_ was often lower than *H*_E_. Following Bonferroni corrections, significant genotypic deviations from HW expectations were observed at eight loci in male and female groups and seven in spermatophores. MICRO-CHECKER v 2.2.3 revealed these significant differences were likely due to the presence of null alleles, but some biological processes also produced positive *F*_IS_ values (e.g.: Wahlund effect from high migration rates). At loci where null alleles were detected, estimated frequencies ranged from 0.1017 at the Aa681 locus in the spermatophores group to 0.3572 at the Aa421 locus in males (Table 2). This latter locus (Aa421) was discarded from all subsequent analyses because its estimated null allele frequency was higher than 0.25 for the three groups.

**Table 2:** Summary statistics of genetic diversity in females, males, and spermatophores of *A*. *antennatus*.

Sample		Aa138	Aa1255	Aa956	Aa496b	Aa123	Aa681	Aa667	Aa1444	Aa751	Aa818	Aa1061	Aa1195	Aa421	All loci
Females	*N*_A_	18	10	6	2	5	15	4	8	3	5	9	4	6	7.3080
	*H*_O_	0.7885	0.4444	0.5192	0.0962	0.4706	0.4510	0.3269	0.4286	0.1346	0.2745	0.3725	0.5962	0.2000	0.3939
	*H*_E_	0.9097	0.7818	0.5907	0.0924	0.6202	0.6992	0.6667	0.7366	0.2285	0.6524	0.7063	0.5913	0.7247	0.6116
	HWE *P*	0.0164	0.0000^*†^	0.1372	1.0000	0.0476	0.0000^*†^	0.0000^*†^	0.0000^*†^	0.0002^*†^	0.0000^*†^	0.0000^*†^	0.9588	0.0000^*†^	
	Nu	0.0632	0.1878	0.0148	0.0000	0.0803	0.1617	0.1981	0.1735	0.0941	0.2333	0.1958	0.0000	0.3004	
	*F*_IS_	0.1333	0.4315	0.1210	−0.0408	0.2412	0.3550	0.5096	0.4182	0.4109	0.5792	0.4725	−0.0083	0.7240	0.3559
	*PI*	0.8220	0.9145	0.7825	0.1705	0.7896	0.8814	0.8101	0.8870	0.3863	0.8259	0.8587	0.7650	0.8781	0.9999●
Males	*N*_A_	18	9	5	2	4	19	7	9	2	6	9	5	7	7.846
	*H*_O_	0.8475	0.4074	0.5085	0.1186	0.5593	0.5763	0.4407	0.3684	0.0678	0.3276	0.4746	0.5254	0.1111	0.4161
	*H*_E_	0.9090	0.6805	0.6479	0.1125	0.6268	0.8013	0.6895	0.7362	0.2624	0.5549	0.7123	0.5809	0.7389	0.6166
	HWE *P*	0.0118	0.0000^*†^	0.0107	1.0000	0.1945	0.0000^*†^	0.0000^*†^	0.0000^*†^	0.0000^*†^	0.0000^*†^	0.0000^*†^	0.1082	0.0000^*†^	
	Nu	0.0413	0.1649	0.0982	0.0000	0.0445	0.1197	0.1534	0.2053	0.1869	0.1568	0.1396	0.0444	0.3572	
	*F*_IS_	0.0677	0.4013	0.2152	−0.0545	0.1077	0.2808	0.3609	0.4996	0.7416	0.4096	0.3337	0.0956	0.8496	0.3251
	*PI*	0.9825	0.8467	0.8262	0.2045	0.7909	0.9440	0.8377	0.8953	0.4168	0.7584	0.8658	0.7484	0.8774	0.9999●
Spermatophores	*N*_A_	19	9	5	2	6	19	6	9	2	7	11	5	7	8.231
	*H*_O_	0.8475	0.4717	0.5893	0.0492	0.7705	0.6230	0.6167	0.4386	0.1967	0.3607	0.4590	0.4068	0.2456	0.4668
	*H*_E_	0.8980	0.6776	0.6503	0.0484	0.6433	0.8219	0.7113	0.6510	0.3579	0.6342	0.7165	0.5869	0.6751	0.6190
	HWE *P*	0.0081	0.0000^*†^	0.3775	1.0000	0.0927	0.0000^*†^	0.0055	0.0009^*†^	0.0011^*†^	0.0001^*†^	0.0000^*†^	0.0168	0.0000^*†^	
	Nu	0.0206	0.1318	0.0151	0.0000	0.0000	0.1017	0.0673	0.1246	0.1333	0.1581	0.1358	0.1123	0.2600	
	*F*_IS_	0.0563	0.3039	0.0939	−0.0169	−0.1977	0.2420	0.1330	0.3263	0.4504	0.4313	0.3594	0.3069	0.6362	0.2459
	*PI*	0.9789	0.8604	0.8222	0.0925	0.802	0.9529	0.8561	0.8080	0.5197	0.8118	0.8864	0.7478	0.8462	0.9999●

*N*_A_, Number of alleles; *H*_O_, observed heterozygosity; *H*_E_, expected heterozygosity; HWE *P*, conformance to Hardy-Weinberg equilibrium; Nu, null allele frequency; *F*_IS_, Wright’s fixation index; *PI*, Probability of Identity; *Significant departure from HWE; ^†^Null alleles detected; ● see text.

**Figure 1:**
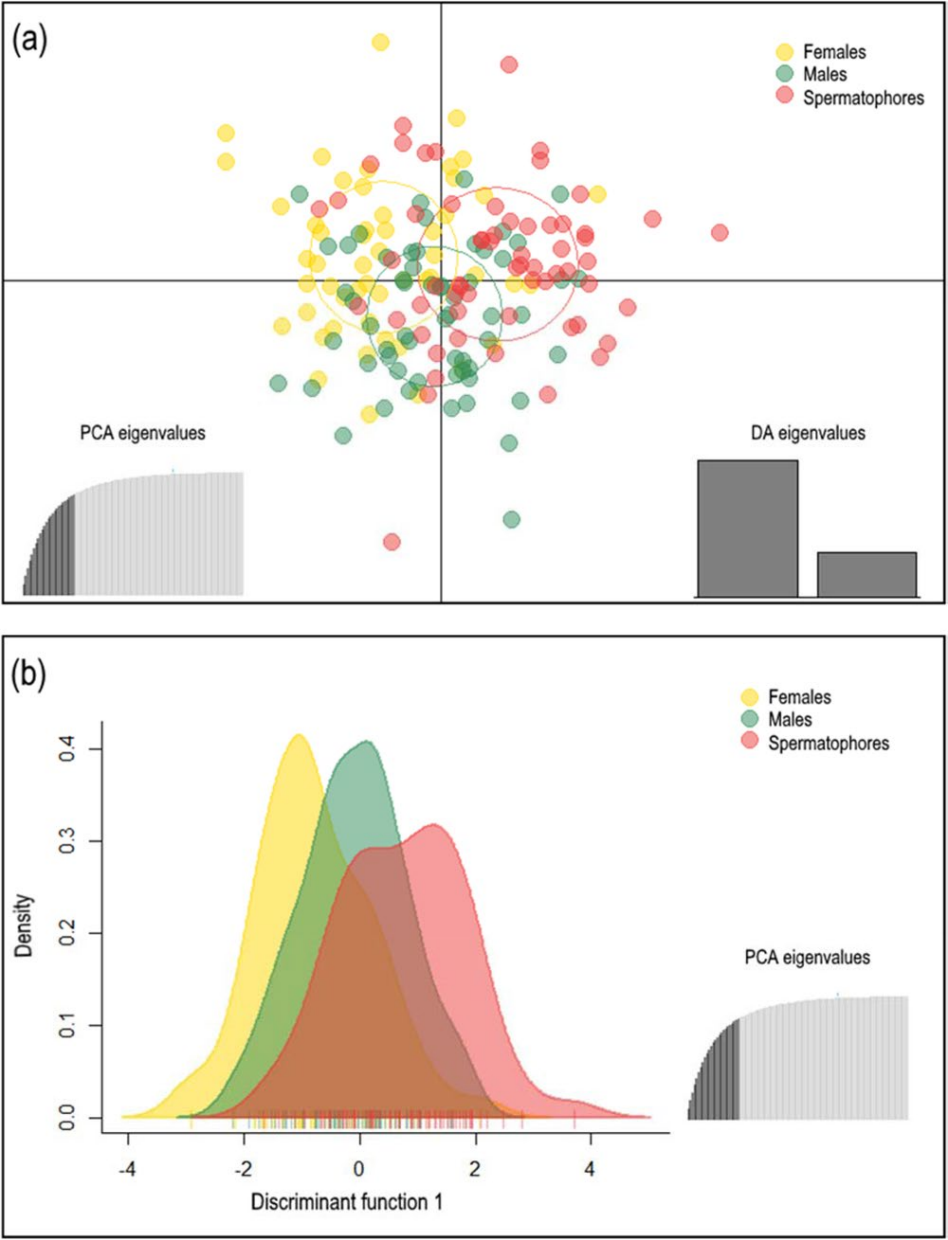
Discriminant Analysis of Principal Components (DAPC) among females, males, and spermatophores. (**a**) Genetic differentiation among females, males, and spermatophores by DAPC. Groups are displayed by different colours and inertia ellipses. Dots represent individuals. The relative portions of the variance captured by first and second axes are illustrated in the DA eigenvalues plot (bottom right corner). The PCA eigenvalues plot in the bottom left corner shows the portion of variance retained when maintaining 25 PCA axes. (**b**) Density plot of individuals along the first discriminant function from the DAPC for females, males, and spermatophores.

Significant allele frequency differences were not detected between males and females (*F*_ST_ = −0.0011; *P* = 0.5000) and males and spermatophores (*F*_ST_ = 0.0016; *P* = 0.5500). Results did detect significant differentiation between females and spermatophores (*F*_ST_ = 0.0091; *P* = 0.0167). Bidimensional DAPC scatterplots obtained from the 25 principal components retained more than 80% of the total genetic variance (Fig. 1a). The first component in the scatterplot depicted a distinction between females and their spermatophores. Males were located in a central position with an overlapping distribution at the left side of the central plot axis scattered with a portion of the females to the right of the main axis with some spermatophores. Spermatophores showed a somewhat bimodal distribution, scattered with females and males for the first component (Fig. 1b).

### Paternity and mating system

Excluding locus Aa421, a combined probability of identity exclusion higher than 0.9999 was observed in all three groups; males, females, and mating males represented by their spermatophores (Table 2). Therefore, the probability that two randomly selected individuals matched their genotypes by chance was lower than 0.0001. In order to check how null alleles could influence probability of identity we conducted an analysis with six loci (Aa138, Aa956, Aa496b, Aa123, Aa751, and Aa1195). We observed the same results we previously generated, suggesting null alleles have no influence on paternity. Multiple spermatophores were identified in six of fifty-two females (11.5%). Three females carried two spermatophores in their thelycum and another three female individuals carried three. In all cases, multiple spermatophores from the same female showed different genotypes. These results indicated multiple mating in females. Identity analysis on 12 microsatellite loci resulted in identical genotypes in one pair of spermatophores from different females, which was consistent with the above estimated exclusion probability of identity, also supporting multiple mating in males. A genetic identity match was not found between spermatophores and males.

All relatedness coefficients produced small and similar estimates between simulated first and second cousin pairs, and more importantly, these values were not clearly differentiated from the values obtained between unrelated pairs of individuals (Table S1). A more robust relatedness estimator was not available for our data set. The triadic likelihood estimator was selected for all further analyses because it typically out performs other estimators in natural populations, where most dyads are unrelated or only loosely related^47^. The pairwise relatedness in our groups ranged from 0.0528 in males to 0.0657 in spermatophores (Table 3). The average relatedness among female individuals (0.0584) was not statistically different from the 1,000 simulated unrelated individuals (0.0591). Similar results were obtained for males, but the observed relatedness among spermatophores differed significantly from the simulated, indicating the spermatophores from males were more closely related than expected by chance alone (Table 3). The average relatedness among females (0.0584) was not significantly higher than the average relatedness among males (0.0528) (Table 3), but relatedness among spermatophores (0.0657) was higher than between all female pairs and male pairs (*P* < 0.01). In addition, the average relatedness between a female-spermatophore pair was similar to all other female-spermatophore pairs, but the value was slightly differentiated from all possible pairs involving a female and an adult male, suggesting some mating males were differentiated from the sampled males (*P* < 0.1) (Table 3).

**Table 3:** Bootstrap test to compare relatedness ± standard error (SE) between groups using TrioML estimator.

Group 1	Mean group 1 ± SE	Group 2	Mean group 2 ± SE	*P*
Females	0.0584 ± 0.0024	Unrelated	0.0591 ± 0.0001	NS
Males	0.0528 ± 0.0021	Unrelated	0.0591 ± 0.0001	NS
Spermatophores	0.0657 ± 0.0023	Unrelated	0.0591 ± 0.0001	***P*** < **0.01**
Females	0.0584 ± 0.0024	Males	0.0528 ± 0.0021	NS
Females	0.0584 ± 0.0024	Spermatophores	0.0657 ± 0.0023	***P*** < **0.01**
Males	0.0528 ± 0.0021	Spermatophores	0.0657 ± 0.0023	***P*** < **0.01**
Females-spermatophore	0.0368 ± 0.0070	All other female-spermatophore pairs	0.0534 ± 0.0016	NS
Females- spermatophore	0.0368 ± 0.0070	All female-male pairs	0.0565 ± 0.0016	0.05 < *P* < 0.1

NS, no significant difference.

## Discussion

The results of our study showed a mean number of alleles per locus (9.38) (Table 2) in *A*. *antennatus* lower than the value reported in Italian blue and red shrimp samples (13.5)^48^. However, our results were consistent with those reported for other shrimp species, including whiteleg shrimp (*Litopenaeus vannamei* Boone, 1931; mean: 7.9)^49^ and the deep-sea hydrothermal vent shrimp (*Rimicaris exoculata* Williams and Rona, 1986; mean: 8.73)^50^. Cannas *et al*.^48^ reported higher mean *H*_O_ (0.65) with an increased number of alleles compared with our data (mean females: 0.39; mean males: 0.42). In both studies, deviations from HW genotype expectations often resulted from heterozygote deficits in several microsatellite loci, a common result in the species of Penaeoidea^51–53^. Several explanations for the pattern have been proposed, including stutter bands, null alleles, and biological factors, e.g. mating tactics, recent population bottlenecks, and/or subpopulation structure. In fact, Wahlund effects resulting from subpopulation structure could not be rejected, as indicated by DAPC results on sampled spermatophores (Fig. 1b). Our results provided clear evidence for the *A*. *antennatus* mating system involves multiple mating for both sexes, suggesting that the reproduction in *A*. *antennatus* involved polyandry (11%) and polygyny (1.5%). We confirmed female multiple mating by conducting an extended genotyping of spermatophores from 20 additional females collected from the same spawning ground in 2016. Each female carried more than one spermatophore in her thelycum and in each sample different male genotypes were obtained (Table S2). Multiple female mating (polyandry) is common across crustacean species, particularly in species where females do not store sperm in specific structures^54^. Multiple mating in invertebrate females: (i) facilitates retention of genetic diversity and increased offspring fitness (e.g., the Pacific gooseneck barnacle^55^); (ii) prevents the risks of choosing an infertile partner (e.g., polychaetes^56^); (iii) favors the storage of enough sperm to fertilize the entire clutch (e.g., lepidopterans^57^); (iv) and might prevent mate competition that causes injuries (e.g., the common rock shrimp^6^). In the case of *A*. *antennatus*, the benefits of multiple mating could be a compendium of the first three factors, due to the high fecundity of females (≅600,000 oocytes produced in each vitellogenesis^26^); the latter option is unlikely due to the sex ratio of the species (females seems to be much more abundant than males^23^) and the small size and without hypertrophied weaponry of the males (major chelipeds and third maxillipeds are evident in aggressively dominant males^58^). Nevertheless, multiple mating does not always translate into multiple paternity. For instance, females of some crustacean species show behavioral and physiological mechanisms to control male fertilization, such as selective sperm passage or spermatophore removal^59,60^. In the caridean rock shrimp (*Rhynchocinetes typus* H. Milne Edwards, 1837), females accept multiple mating to avoid injury by harassing males, but afterwards remove spermatophores from subordinate males to guarantee sperm from the highest quality males^61,62^. Thus, our *A*. *antennatus* findings demonstrated multiple mating, but did not ensure multiple paternity.

Our data demonstrated that males and females were not genetically differentiated, but mating males, inferred from spermatophores, were somewhat distinct from sampled males. In fact, the DAPC scatterplots showed a portion of spermatophores did not overlap with adult males (Fig. 1b) and females were clearly differentiated from their spermatophores. Females apparently bias mating towards unrelated males. Mating males inferred from spermatophores might include those sympatric with females, as well as those from other spawning grounds. Evidence for mate choice in favor of genetically dissimilar partners was reported in some invertebrates, interpreted as a way of inbreeding avoidance, which improves reproductive survival and individual fitness, and increases genetic diversity of local populations^63^, and references therein. Liu *et al*.^64^ found that females of the cabbage beetle (*Colaphellus bowringi* Baly, 1865) preferred to mate with non-siblings rather than siblings, decreasing the potential for inbreeding depression. Simmons *et al*.^65^ observed similar behavior in the Australian black field cricket (*Teleogryllus oceanicus* Le Guillou, 1841), where females fertilized their eggs with sperm from non-sibling males rather than full-siblings. Gherardi *et al*.^66^ suggested that invertebrates make genetic decisions regarding their mating partners; however, relatively little is known about the mechanisms by which recognition is performed, especially in deep-water species, which are not amenable to laboratory conditions, as is the case for *A*. *antennatus*. One possibility for the observations made is that these species, use waterborne chemical cues (olfactory) to obtain information about conspecifics and consequently distinguish between kin and non-kin individuals^67^. The big-clawed snapping shrimp (*Alpheus heterochaelis* Say, 1818) employs this recognition mechanism to differentiate between familiar and unfamiliar individuals^67^. Another possibility is these taxa develop an olfactory and contact chemoreception combination mechanism, comparable to the eusocial coral-reef shrimp (*Synalpheus regalis* Duffy, 1996), where colony members discriminate between nest-mates and foreign conspecifics^19,68^. Often, marine Penaeoidean shrimps with pure searching mating strategy have small sized males, as does *A. antennatus*, and use contact chemical communication to search for receptive females in reproductive aggregates^21–23,58^. This strategy, for us the most plausible in *A. antennatus* was observed in terrestrial (e.g., mangrove tree crab^69^) and freshwater decapoda (e.g., freshwater atyid shrimp^70^).

In general, when individuals of one sex do not disperse far from the natal group, they are expected to have increased relatedness on average than those that disperse^71^. Our results showed that the spermatophores were differentiated from sampled males and females, suggesting that they were deposited in the female thelycum by *A*. *antennatus* migrating males. In contrast, using different set of loci Cannas *et al*.^48^ analyzed the populations of *A*. *antennatus* of three Italian collections at different depths (Sant’ Antioco, <800 m; Pesca Profonda-Sperimentale North, 1,464 m; Pesca Profonda-Sperimentale South, 1113 m) and suggest female-biased dispersal. The different depth sampling design might explain the lack of congruency between Cannas *et al*.^48^ and present results (600 m). Oceanographic currents in the Mediterranean Sea are different among water masses at distinct depths (Surface Waters, <150 m; Intermediate Waters, 150–1,000 m; Deep Waters, >1,000 m) which could facilitate different dispersal patterns^72^. Contrasting results on dispersal patterns might also reflect temporal and spatial ecological variation affecting resource availability and population density^21–23,73,74^. Otherwise, the sex ratio obtained in Pesca Profonda-Sperimentale North (1:4) differed from that in the North - Western Mediterranean Sea^23,26,73^, which suggested that the former locality supports a unique population dynamic.

Our research demonstrated a polyandry and polygyny mating system in the blue and red shrimp. Additional research in different natural populations will be fundamental to fully elucidate the mating system in *A*. *antennatus*. Mating tactics might vary among populations depending on population density, mate quality, mate availability or variation in environmental conditions, as Gosselin *et al*.^75^ reported in the American lobster (*Homarus americanus*). The apparent capacity of *A*. *antennatus* to select mating partners based on genetic dissimilarity provides the opportunity to pursue new research avenues and explore possible factors influencing mate choice in this species. Additionally, it will be important to include information regarding life history variation and strategies for stock assessment and effective fishery management of heavily exploited marine resources, such as *A*. *antennatus*, to ensure sustainability.

## Supplementary information

Supplementary information

## Data Availability

All data are included in the article.
