# Supplementary material for: Mating structure of the blue and red shrimp, Aristeus antennatus (Risso, 1816) characterized by relatedness analysis
Source: Sci Rep. 2019 May 10;9:7227. doi: 10.1038/s41598-019-43523-w (PMC6510731; doi:10.1038/s41598-019-43523-w)
Supplement: Supplementary file 1 — Supplementary information [file 41598_2019_43523_MOESM1_ESM.pdf]

Supplementary information for

Mating structure of the blue and red shrimp, *Aristeus antennatus* (Risso, 1816) characterized by relatedness analysis

Laia Planella[1], Manuel Vera[1,2], Jose-Luis García-Marín[1], Sandra Heras [1]*, María Inés Roldán[1]

[1] *Laboratori d'Ictiologia Genètica, Universitat de Girona, Girona,* Spain.

[2] *Present address: Departamento de Zooloxía, Xenética e Antropoloxía Física, Campus Lugo, Universidade de Santiago de Compostela, Lugo, Spain.*

*Corresponding author: sandra.heras@udg.edu

This pdf file includes:

Table S1.

Table S2.

Table S3.

Table S1. Mean relatedness ± variance for seven relatedness estimators for a simulated population consisting of 1,000 pairs of unrelated, second cousin, and first cousin pairs with allele frequencies obtained from field data.

Relationship	r_{xy}	TrioML	Wang	LynchLi	LynchRD	Ritland	QuellerGt	DyadML
Unrelated	0	0.0528 ± 0.0073	- 0.0070 ± 0.0362	- 0.0052 ± 0.0414	- 0.0006 ± 0.0118	- 0.0006 ± 0.0204	- 0.0006 ± 0.0349	0.0712 ± 0.0111
Second Cousins	0.0312	0.0527 ± 0.0073	- 0.0084 ± 0.0364	- 0.0057 ± 0.0415	- 0.0006 ± 0.0119	- 0.0006 ± 0.0209	- 0.0006 ± 0.0343	0.0711 ± 0.0111
First cousins	0.1250	0.0522 ± 0.0072	- 0.0100 ± 0.0354	- 0.0077 ± 0.0403	- 0.0006 ± 0.0118	- 0.0006 ± 0.0207	- 0.0006 ± 0.0341	0.0704 ± 0.0109

r_{xy}: expected relatedness value.

Table S2. Microsatellite profiles of 20 females and their pair of spermatophores removed from female thelycum. Female, spermatophore 1 (Sperm1) and spermatophore 2 (Sperm 2) genotypes for Aa138 and Aa681 microsatellite loci. -/-: No genotype.

	**Locus Aa138**			**Locus Aa681**		
**Specimen**	**Female**	**Sperm 1**	**Sperm 2**	**Female**	**Sperm 1**	**Sperm 2**
Aa2682	213/213	217/217	213/213	236/236	236/236	240/240
Aa3578	195/201	207/209	205/231	230/322	248/248	230/238
Aa3587	213/221	191/191	201/201	322/322	236/236	236/236
Aa3596	197/207	201/201	205/217	236/260	242/242	238/264
Aa3604	231/231	211/213	201/207	236/236	262/262	224/224
Aa3619	201/223	201/201	201/201	236/238	262/262	238/238
Aa3640	203/213	199/199	205/205	264/270	236/236	264/264
Aa3662	187/207	217/217	201/201	236/238	236/236	236/236
Aa3706	201/221	213/213	197/199	238/238	236/236	236/236
Aa2	-/-	187/223	211/231	-/-	240/240	240/240
Aa3	-/-	195/213	203/217	-/-	236/260	286/286
Aa6	-/-	213/217	201/217	-/-	236/292	236/236
Aa7	-/-	213/213	207/207	-/-	238/238	240/240
Aa9	-/-	195/217	211/225	-/-	236/236	262/270
Aa12	-/-	205/205	221/221	-/-	260/260	236/236
Aa13	-/-	199/199	191/229	-/-	320/320	264/264
Aa18	-/-	213/213	217/217	-/-	264/264	224/224
Aa19	-/-	207/231	213/231	-/-	236/250	232/236
Aa22	-/-	219/223	205/217	-/-	230/248	236/236
Aa24	-/-	193/213	191/225	-/-	236/244	242/292

Table S3. Raw data set. Female (F), male (M), spermatophore (E).

Name	Aa138A	Aa138B	Aa1255A	Aa1255B	Aa956A	Aa956B	Aa496A	Aa496B	Aa123A	Aa123B	Aa681A	Aa681B	Aa667A	Aa667B	Aa1444A	Aa1444B	Aa751A	Aa751B	Aa818A	Aa818B	Aa1061A	Aa1061B	Aa1195A	Aa119B	Aa421A	Aa421B
F1104	201	223	137	139	193	213	412	412	429	431	236	260	253	253	182	182	225	235	165	165	191	197	195	201	181	193
F1105	213	217	125	137	213	213	412	412	431	431	236	236	253	256	182	184	230	230	170	170	191	191	198	198	185	185
F1106	201	213	125	125	201	209	412	412	431	431	236	264	250	250	184	184	230	230	170	190	191	197	198	201	185	185
F1107	213	221	137	155	213	213	412	418	427	429	236	270	253	253	182	194	230	230	180	180	195	195	195	198	181	181
F1108	199	219	125	125	213	213	412	412	431	431	236	238	256	256	182	182	230	230	170	170	191	207	195	195	185	185
F1109	201	201			213	213	412	412			236	258	256	256			230	230	175	175	197	207	195	195	181	181
F1110	205	219			213	213	412	412	427	431	236	264	253	253	194	194	230	230	170	170	191	191	195	195	189	191
F1111	213	223	125	139	209	213	412	412	427	429	236	236	256	256	182	192	230	230	170	170	191	191	195	201	185	187
F1112	205	217	139	139	209	213	412	412	427	429	236	236	250	250	182	184	230	230	190	190	191	207	195	201	189	189
F1113	201	213	133	133	205	209	412	412	427	431	238	238	250	250	192	202	230	230	170	170	207	207	195	198	185	185
F1114	205	219	125	133	213	213	412	412	427	427	236	236	253	253	182	182	230	230	165	170	207	207	198	198	185	185
F1115	195	195			205	209	412	412	429	431	236	238	250	256			235	235	170	170	191	205	195	198	185	185
F1116	221	221	139	139	205	209	412	412	427	431	236	236	250	256	182	182	230	230	175	175	189	189	195	195		
F1117	201	217	135	137	213	213	412	412	429	429	236	236	250	253			230	230	170	170	191	195	195	198	189	189
F1118	201	227	137	137	213	213	412	412	431	431	236	240	256	256	182	184	225	225	170	170	143	191	195	198		
F1119	199	219	125	137	209	213	412	412	431	431	240	240	253	253	182	182	230	230	170	170	207	207	195	198	189	189
F1120	207	213	133	137	213	213	412	412	427	429	236	240	256	256	182	194	230	230	190	190	207	207	198	201	185	187
F1121	217	231	125	139	205	209	412	412	427	427	236	314	250	256	184	188	230	230	170	170	191	207	195	195	185	185
F1122	213	221			213	213	412	412	431	431	236	236	256	256	182	182	230	230	170	170	191	207	195	195	185	185
F1123	201	201	137	137	213	213	412	412	431	431	236	240	253	253	184	194	230	235	175	175	207	207	195	195	189	189
F1124	203	243	155	155	209	213	412	412	427	431	238	238	250	256	182	192	230	230	170	170	191	191	195	198	187	187
F1125	221	231	137	137	201	209	412	412	431	431	236	238	253	256	194	194	230	230			207	207	195	195	185	187
F1126	201	227	125	125	213	213	412	412	431	431	236	236	250	253	182	194	230	235	170	170	195	195	195	195	187	187
F1127	221	221	125	125	209	209	412	412	429	431	236	236	250	250	192	194	230	230	170	170	191	207	195	195	181	187
F1128	201	201			209	213	412	412	431	431	236	264	244	256	182	182	230	230	170	170	207	207	195	201	185	187
F1129	211	217	139	139	213	213	412	412	427	427			250	253	192	194	230	235	170	180	189	207	198	198	189	189
F1130	201	217	141	141	213	213	412	412	431	431	238	238	250	250	182	182	230	230	165	175	191	191	195	201	185	185
F1132	201	213	137	155	213	213	412	412	429	429	320	320	250	250	194	194	230	230	170	170	195	195	195	195	185	185
F1133	201	203	137	137	209	213	412	412	427	429	224	236	256	256	180	184	230	230	175	175	191	191	198	201	185	185
F1134	213	231	137	137	205	209	412	412	427	429	230	236	250	256	182	192	230	230	165	170	191	207	195	198		
F1135	217	227			213	217	412	412	431	431	236	236	253	256	194	194	230	230	170	175			198	201	185	185
F1136	195	211	137	137	205	213	412	412	427	431	270	270	256	256	182	182	230	230	170	175	197	207	195	201	185	185
F1137	191	197	139	139	213	213	412	412	425	431	236	260	250	250	194	194	230	230	175	175	191	191	195	201	187	187
F1138	213	217	123	123	213	213	412	418	431	431	236	264	253	256	182	194	230	230	180	180	207	207	195	201	185	185
F1139	187	221	155	163	213	213	412	412	427	431	236	236	250	250	182	182	230	230	175	190	207	207	195	198	185	185
F1141	201	219	137	155	209	213	412	412	431	431	236	236	250	253	188	188	230	230	170	170	189	193	195	195	185	185
F1142	201	217	137	137	213	213	412	412	431	433	238	238	250	253	182	182	230	230	165	170	207	207	198	201	181	181
F1143	201	205	137	141	201	213	412	412	429	431	236	236	250	250	184	194	225	235	170	170	189	191	201	201	191	191
F1145	203	203	125	139	201	209	412	412	429	429	230	238	256	256	192	192	230	230	170	170	207	207	195	201	187	187
F1146	199	211	139	139	205	205	412	412	431	431	260	260	250	250	182	184	230	230	175	175	191	191	195	198		
F1147	227	231	137	137	205	213	412	412	427	427	236	262	250	250	182	186	230	230	175	175	191	205	195	198	181	189
F1148	213	213	137	139	213	213	412	418	427	429	236	322	250	250	182	182	230	230	175	175	191	191	195	195	189	189
F1149	197	197	125	125	205	205	412	412	427	431	236	264	256	256	194	194	230	230	165	170	191	191	195	195		
F1150	201	213	125	125	205	213	412	412	431	433	236	236	250	253	194	194	230	230	165	165	205	205	198	201	187	187
F1151	201	211	135	137	205	213	412	418	431	431	238	238	256	256	182	184	230	235	165	165	207	207	195	198		
F1152	213	213	137	139	213	213	412	412	427	431	236	236	250	250	184	184	230	230	170	170	191	191	195	201	181	181
F1153	213	217	125	125	209	213	412	412	429	431	238	240	253	253	188	188	230	235	165	170	207	207	195	198	185	189
F1154	203	213			205	213	412	412	431	431	238	238	250	250	182	182	230	230	175	175	207	207	192	195	185	185
F1155	205	211	137	143	209	213	412	412	427	427	236	236	250	256	182	186	230	230	170	175	191	207	195	195		
F1156	211	227	125	139	201	205	412	412	431	431	236	248	256	256	182	182	230	230	165	170	191	207	195	195	185	185
F1157	231	231	137	137	213	213	412	418	431	431	264	264	256	256	184	184	230	230	170	175	209	209	195	198	191	191
F1158	203	231	125	137	209	213	412	412	427	431	244	244	250	250	184	184	230	230	170	170	207	207	195	195	189	189
M1104E	201	217	133	137	213	213	412	412	429	431	248	322	250	256	194	200	230	230	175	175	191	191	195	195	185	185
M1105-1E	191	231	137	137	213	213	412	412	429	431	236	236	244	250	184	192	230	235	170	170	195	207	195	198	185	185
M1105-2E	215	219					412	412	429	431	236	270	244	244	182	194	230	235	175	175	207	207	195	195	187	187
M1106-1E	203	217			209	213	412	412	429	431	236	258	250	256	182	194	230	230	165	175	193	207	195	198	185	185
M1106-2E	199	199	137	139	209	213	412	412	429	431	262	264	244	250	194	194	235	235	170	175	185	193	195	195	181	185
M1107E	203	213	137	137	213	213	412	412	427	427	236	262	250	250	182	182	235	235	170	170	191	191	195	195	187	187
M1108E	213	213	125	139	209	213	412	412	429	431	236	264	253	256	186	194	235	235	170	170	197	207	195	198	187	187
M1109-1E	213	221	125	137	201	209	412	412	429	431	236	264	250	250	182	194	230	230	170	170	207	207	195	198		
M1109-2E	223	223	125	137			412	412	429	431	236	236	250	256	182	188	230	230	170	175	191	207	195	198	185	185
M1109-3E	199	217	137	137			412	412	429	431	236	236	250	256	182	182	230	230	170	170	207	211			185	185
M1110E			137	137	213	213	412	412	429	431	230	236	253	256	194	194	230	235	170	175	207	207	198	198	185	187
M1111E	217	221	137	137	213	213	412	412	429	431	232	260	253	256	192	192	230	230	170	170	191	191	195	195	185	189
M1112E	195	217	137	137	193	209	412	412	427	431	264	322	253	253	194	194	230	230	175	185	207	207	195	195	187	187
M1113-1E	193	201	125	137	205	213	412	412	431	431	240	314	256	256	182	182	230	230	170	170	143	197	195	195	181	181

M1113-2E	201	205	125	137	205	213	412	412	431	431	268	268	250	250	182	182	230	230	165	210	195	197	195	198	185	185
M1113-3E			133	133			412	412	427	429	236	236	253	253	182	194	230	230	170	175	191	209			185	185
M1114E	187	213	139	139	201	213	412	412	429	431	236	264	250	250	182	182	230	235	170	170	195	207	198	198	185	185
M1115E	195	205	137	137	205	205	412	412	427	429	236	236	250	253	182	182	230	230	175	190	207	207	201	201	187	187
M1116E	201	205	137	137	209	213	412	418	429	431	258	258	241	250	194	194	230	230	170	170	191	195	195	195	185	185
M1117E	217	231			205	209	412	412	429	431	236	264	253	256	182	194	230	235	170	170	191	207	198	198	185	185
M1118E	207	217	137	137	213	213	412	412	427	431	236	260	250	253	182	182	230	235	175	175	191	197	195	198	185	189
M1119E	207	225	125	137	213	213	412	412	429	431	236	236	250	250	182	194	230	230	175	175	207	207	195	198	181	181
M1120E	203	217	137	139	213	213	412	412	427	431	240	270	250	250	194	194	235	235	170	170	191	191	198	198	185	187
M1121E	201	207	139	141	209	213	412	412	427	431	236	236	250	256	182	182	230	230	175	190	191	209	198	198	181	187
M1122E	213	213	137	139	213	213	412	418	427	431	236	266	253	256	182	182	230	230	175	190	189	207	195	195		
M1123E	201	213	125	125	205	213	412	412	431	431	230	230					230	230	170	175	207	207	195	198	189	189
M1124E	217	221	125	137	205	205	412	412	431	431	236	236	253	256	182	194	230	235	165	175	207	207	198	198		
M1125E	199	217	137	137	201	213	412	412	431	431	230	236	256	256	192	194	230	235	165	165	191	207	195	198	187	187
M1126E	201	231	125	125	209	213	412	412	429	431	238	264	250	256	180	192	230	230	170	170	207	207	198	201	189	189
M1127-1E	213	217	139	139	213	213	412	412	427	427	236	236	253	256	182	198	235	235	170	170	207	207	198	201	185	185
M1127-2E	213	217	125	137	213	213	412	412	427	431	236	260	256	256	194	194	230	230	170	170	207	207	195	195	185	187
M1127-3E	213	213	137	141	201	213	412	412	427	429	236	236	253	256	182	194	230	230	170	170	191	195	195	201	185	185
M1128E	195	205	137	137	205	205	412	412	427	429	236	236	250	253	182	182	230	230	175	190	207	207	201	201	187	187
M1129E	199	199	123	141	201	205	412	412	429	431	264	264	250	256	182	194	230	230	170	170	205	207	195	195	187	207
M1130E	195	205			213	213	412	412	431	431	240	240	250	256	194	194	230	235	170	170	207	207	198	198	189	189
M1132E	205	217	137	137	213	213	412	412	427	431	264	322	250	253	182	194	235	235	180	180	191	191	198	198	185	189
M1133E	213	217	137	137	205	213	412	412	429	431	230	278	250	250	194	198	235	235	170	170	189	189	198	198	193	193
M1134E	207	217	133	137	213	213	412	412	429	429	264	322	253	256	194	194	230	235	170	170	207	207	195	201	187	187
M1135E	213	221	137	137	201	205	412	412	429	429	266	266	253	253			230	235	170	170	195	195	192	195	185	185
M1136-1E	195	213	125	137	201	209	412	412	429	429	236	322	250	256	182	182	230	230	170	175	207	207	195	195	185	185
M1137E	201	213	137	137	213	213	412	412	427	431	238	250	256	256	184	194	230	230	170	170	191	191	195	204	187	187
M1138E	199	205					412	412	427	431	236	236	256	256	182	192	230	230	175	175	207	207	198	198	185	185
M1139E	217	219	125	137	209	213	412	418	431	431	250	264	250	253	194	198	230	230	170	170	207	207	195	201	187	187
M1141E	217	219	137	137	205	213	412	412	431	431	260	264	256	256	194	194	230	230	165	175	207	207	195	195		
M1142E	213	223	125	125	201	205	412	412	427	429	236	264	250	256	182	194	230	230	170	170	197	207	195	195	185	185
M1143E	199	201	135	139	205	213	412	412	429	431	238	282	250	256	182	194	230	230	170	170	195	197	195	201	185	185
M1145E	203	231			205	209	412	412	431	431	236	282	250	256			230	230	165	170	195	195	195	195	185	195
M1146E	211	213	137	137	205	213	412	412	429	431	230	230	253	253			230	230	170	175	195	195	198	198	185	185
M1147-1E	207	231	135	143	209	213	412	412	429	431	238	248	250	256	198	198	230	235	170	170	191	209	195	195	185	187
M1147-2E	195	217	137	137	213	213	412	412	429	431	238	238	250	253	194	194	230	230	165	170	205	207	195	198	185	185
M1148E	213	225	125	137	205	213	412	412	429	431	238	264	250	256	182	182	230	230	175	175	207	207	195	198	189	189
M1149E	217	231	133	137	205	209	412	412	431	433	228	248	250	253	182	182	230	230	170	185	191	209	195	201	185	189
M1150E	213	213			205	205	412	412	427	431	228	314	244	244	182	182	230	230	170	170	191	207	195	198	185	185
M1151E	201	227	135	135	205	213	412	412	429	431	236	236	253	256	194	194	230	230	175	175	205	205	195	195	185	185
M1152E	191	199	125	125	205	205	412	412	429	431	236	238	256	256	182	182	230	230	165	170	191	197	195	195	181	181
M1153E	205	211	137	141	209	209	412	412	425	427	238	270	256	256	182	182	230	230	170	170	197	207	195	198	195	195
M1154E	201	231	115	115	213	213	412	412	427	429	264	264	253	256	182	194	235	235	180	180	207	207	195	195	185	193
M1155E	213	213			209	213	412	412	431	435	230	236	250	253	192	192	230	230	175	180	189	207	195	195	185	185
M1156E	201	217	139	139	209	213	412	412	429	431	236	266	244	262	182	194	230	230	175	175	207	207	195	195	189	189
M1157E	201	205	137	141	205	213	412	412	427	431	236	258	250	250	182	182	230	230	165	165	189	205	195	201	185	185
M1158E	213	213	137	143	209	213	412	412	429	431	236	236	253	253	194	194	230	230	170	190	207	207	198	198	185	189
M1159	195	199			205	213	412	412	427	427	236	264	250	250	182	184	230	235	170	175	191	207	195	195		
M1160	201	217	137	139	213	213	412	412	429	431	236	236	256	256	188	188	235	235			207	207	195	195	189	189
M1161	213	231	137	137	205	209	412	412	431	431	238	266	253	256	182	182	230	230	175	185	197	207	195	195	185	185
M1162	195	201	137	137	213	213	412	412	425	431	236	240	244	244	194	194	230	230	175	175	191	191	195	198	189	189
M1163	217	231	133	137	209	209	412	412	429	429	238	264	241	256	182	182	230	230	165	170	191	207	195	195	185	185
M1164	211	217	125	125	213	213	412	418	429	431	236	322	256	256	186	194	230	230	170	170	207	207	195	195	187	191
M1165	201	233			213	213	412	412	427	429	264	282	256	256	182	194	230	230	170	170	195	207	195	195		
M1166	217	219	137	137	205	213	412	412	429	431	236	236	256	256	182	182	230	230	165	165	191	197	198	201		
M1167	199	199	125	139	205	213	412	418	429	431	230	236	253	256	194	194	235	235	170	170	191	207	195	201		
M1168	219	221	137	137	205	213	412	418	427	431	258	258	253	253	182	198	230	230	170	170	191	191	195	198	185	185
M1169	221	221	133	137	201	213	412	412	429	431	238	238	250	250	192	194	230	230	165	170	191	191	195	198	187	189
M1170	231	231	139	139	205	213	412	412	427	431	236	236	250	256	182	182	230	230	175	175	191	207	195	201	185	185
M1171	197	205	137	137	201	201	412	412	431	431	238	266	253	253	194	194	230	230	190	190	195	197	195	198	185	185
M1173	213	217	139	139	213	213	412	418	429	429	230	240	253	253	182	182	230	230	170	175	191	191	195	198		
M1174	211	217	137	143	213	213	412	412	431	431	236	262	247	247	192	194	230	235	170	180	191	191	195	198	185	187
M1175	201	231	137	137	205	213	412	412	431	431	260	260	250	253	182	182	230	230	175	175	191	207	195	201	185	185
M1176	201	217	125	125	209	209	412	412	429	431	236	236	250	256	182	182	230	230	170	170	195	209	195	201	185	185
M1177	213	217	137	137	201	213	412	412	429	431	236	236	250	253	182	182	230	230	170	170	205	207	195	195	185	185
M1178	231	231	135	137	205	213	412	412	427	427	320	320	250	250	184	192	230	230	170	170	191	191	198	198	189	189
M1179	187	219			209	213	412	412	427	429	240	242	253	253	184	184	230	230	170	170	191	207	195	195	189	189

M1180	201	211	137	137	205	205	412	412	429	431	236	236	250	256	194	194	230	230	170	175	191	191	195	195	185	185
M1181	201	217	125	137	213	213	412	412	431	431	236	238	250	250	182	192	230	230	165	165	195	207	195	195		
M1182	201	211	139	139	201	213	412	412	429	431	236	236	250	250	194	194	230	230	170	170	207	207	195	195	185	185
M1183	195	221	133	137	201	213	412	412	429	431	236	236	250	256	192	194	230	230	170	170	191	207	195	195		
M1184	219	221	133	133	213	213	412	412	427	429	236	282	250	256			230	230	170	170	207	207	195	198	187	187
M1185	211	217	125	125	205	213	412	412	431	431	314	314	256	256	182	182	230	230	170	170	207	207	195	201		
M1186	217	217	137	137	193	193	412	412	427	431	236	260	256	256	192	192	230	230	170	170	195	207	198	198	181	181
M1187	215	229	125	125	209	213	412	418	427	429	238	238	250	253	194	198	230	230	170	170	191	207	195	201	181	181
M1188	199	227	137	137	213	213	412	412	429	431	236	286	250	256	198	198	230	230	165	165	191	207	195	198	185	185
M1189	195	219	115	115	209	209	412	412	429	431	236	258	250	256	184	184	230	230	170	175	189	189	195	201	189	189
M1190	205	211	125	125	209	213	412	412	427	427	236	240	250	250	182	182	235	235	170	175	207	207	198	198		
M1191	201	217	125	137	209	213	412	412	429	431	264	264	253	256	192	192	235	235	170	170	207	207	192	198	187	187
M1192	201	203	125	137	213	213	412	412	431	431	286	286	250	256	182	182	230	230	175	190	205	207	195	198	185	185
M1193	187	217	137	139	205	209	412	412	429	431	230	236	256	256	188	192	230	230	170	170	197	207	198	198	185	185
M1194	201	231	135	141	213	213	412	412	431	431	236	282	256	256	182	194	230	230	170	170	191	207	195	195		
M1195	187	205	125	137	213	213	412	412	429	429	264	264	250	256	202	202	230	230	170	175	191	191	198	204	189	189
M1196	187	219	137	139	213	213	412	412	427	427	236	268	250	250	186	194	230	230	170	170	143	207	195	195		
M1197	201	213	137	137	201	205	412	412	429	429	236	264	256	256	182	182	230	230	175	175	195	197	198	201	181	181
M1198	199	213	137	137	205	209	412	412	425	431	242	270	253	256	182	182	230	230	170	190	209	209	195	198		
M1199	199	217	137	137	193	213	412	412	431	431	236	236	250	256	192	192	230	230	165	170	191	191	195	195	185	185
M1200	203	217	125	137	213	213	412	412	427	431	240	248	253	253	182	192	230	230	175	175	207	207	195	198	169	169
M1201	217	231	125	139	205	205	412	412	431	431	236	262	256	256	182	184	230	230	170	170	191	207	195	195	187	187
M1202	201	205	137	137	209	209	412	412	429	431	238	258	250	256	194	194	230	230	170	170	207	207	198	198	187	187
M1203	213	231	125	163	213	213	412	412	427	429	238	314	256	256	190	194	230	230	170	170	191	191	195	195	187	187
M1204	187	213	125	137	205	213	412	412	431	431	236	236	250	253	182	182	230	235	165	170	191	191	195	198	187	187
M1205	207	217	125	125	205	213	412	412	431	431	236	236	250	250	182	182	230	230	170	170	207	207	198	201	185	189
M1206	201	205	125	137	213	213	412	412	427	429	248	282	256	256	182	182	235	235	170	170	191	191	195	195	187	187
M1207	197	197	125	137	209	209	412	412	429	431	248	262	250	253	184	184	230	230	170	180	207	207	198	198	189	189
M1208	211	211			201	213	412	418	427	431	236	238	250	256	182	192	235	235	165	175	197	197	195	198	185	185
M1209	203	213	139	139	205	213	412	412	427	431	230	236	256	256	182	182	230	230	170	175	207	207	204	204	187	187
M1210	201	217	125	125	205	205	412	412	431	431	236	236	256	256	182	182	230	235	170	170	197	197	195	195	205	205
M1211	221	221	137	137	213	213	412	412	431	431	238	264	250	256			235	235	170	170	143	143	195	198	187	187
M1212	203	211	125	139	213	213	412	412	431	431	236	276	250	250	182	182	230	230	170	170	197	209	195	198	189	189
M1213	213	217			205	213	412	412	427	431	238	238	253	262	182	182	230	230	170	175	197	207	195	198	189	189
M1214	201	201	137	137	205	213	412	418	431	431	260	264	253	256	182	192	230	230	170	170	191	207	195	198	189	189
M1215	217	219	125	137	205	213	412	412	429	431	236	236	250	250	186	192	230	230	170	190	179	197	195	198		
M1216	201	221	137	137	209	213	412	412	427	427	236	236	253	256	182	182	230	230	170	170	191	207	195	195		
M1217	213	217	137	137	213	213	412	412	429	431	236	236	253	253	198	202	230	230	180	180	207	207	195	198	185	187
M1218	207	217	139	139	201	209	412	412	431	431	238	264	253	253	182	184	230	230	170	170	191	191	198	198	185	185
